# The Value of Five Novel Anthropometric Indicators in Evaluating MASLD and Liver Fibrosis in MASLD: A Cross‐Sectional Study

**DOI:** 10.1002/jgh3.70435

**Published:** 2026-07-08

**Authors:** Huiya Huang, Yangni Lu, Tong Luo, Huabei Wu, Jinfeng Li, Tingting Tang, Xianli Xv, Jianlin Wu, Maowei Chen

**Affiliations:** ^1^ Department of General Medicine Wuming Hospital of Guangxi Medical University Nanning Guangxi China; ^2^ Department of Infectious Diseases Wuming Hospital of Guangxi Medical University Nanning Guangxi China; ^3^ Department of Gastroenterology The People's Hospital of Laibin City Laibin Guangxi China; ^4^ Dean's Office Youjiang Medical University for Nationalities Baise Guangxi China; ^5^ School of General Practice Guangxi Medical University Nanning Guangxi China

**Keywords:** anthropometric indices BRI, early identification, MASLD, NHANES LF

## Abstract

**Background:**

Metabolic dysfunction‐associated fatty liver disease (MASLD) is the most prevalent chronic liver disease globally. Early screening is critical for preventing liver fibrosis (LF) and cirrhosis, but liver biopsy and imaging are costly and less accessible in primary care settings. Simple, economical anthropometric indicators are urgently needed for large‐scale initial screening.

**Methods:**

Using data from NHANES 2017–2020, we applied logistic regression, generalized additive models, and ROC analysis to evaluate five novel anthropometric indices (BRI, LAP, WTI, WWI, and ABSI) for predicting MASLD and LF in individuals with MASLD.

**Results:**

All indices showed significant positive dose–response associations with MASLD risk (fully adjusted ORs: BRI, 1.86; LAP, 1.03; WTI, 5.31; WWI, 3.31; and ABSI, 2.13). BRI and LAP demonstrated the highest predictive performance for MASLD (AUC = 0.82). For LF among individuals with MASLD, BRI (AUC = 0.71) outperformed FIB‐4 and APRI. Stratified analyses revealed sex‐, age‐, and BMI‐related differences: WTI performed better in women; ABSI and BRI in men; WWI, ABSI, and BRI in younger adults; and BRI in lean individuals. Nonlinear associations with MASLD were observed for all indices, whereas only WWI showed a linear association with LF.

**Conclusions:**

BRI is independently associated with MASLD and LF in individuals with MASLD and demonstrates superior predictive performance and clinical net benefit compared with other noninvasive models. These findings support its potential use for the early identification of MASLD and LF risk in primary care settings.

**Abbreviations**
ABSI: A Body Shape Index
BRI: body roundness index
CAP: controlled attenuation parameter
HSI: hepatic steatosis index
LAP: lipid accumulation product
LF: liver fibrosis
LSM: liver stiffness measurement
MASLD: metabolic dysfunction‐associated fatty liver disease
WC: waist circumference
WTI: weight‐to‐height index
WWI: weight‐adjusted‐waist index

## 1 Introduction

The liver acts as the central regulator of lipid homeostasis and orchestrates crucial physiological processes, including blood volume regulation, immune system maintenance, and endocrine‐modulated growth signaling. Metabolic dysfunction‐associated fatty liver disease (MASLD), a World Health Organization priority noncommunicable disease [1], threatens global health, with a worldwide prevalence of 1.27 billion in 2021 (a 24.3% increase since 1990) [2, 3]. Liver fibrosis (LF) correlates strongly with poor prognosis in MASLD [4, 5, 6].

Current MASLD diagnosis faces several challenges. While liver biopsy remains the gold standard, its invasive nature carries inherent risks. FibroScan has compromised accuracy in patients with obesity (body mass index [BMI] of > 30 kg/m^2^), ascites, or narrow intercostal spaces [7]. By contrast, although obesity indices cannot directly quantify liver pathology, they offer advantages in terms of simple measurement and low cost, making them more suitable for large‐scale initial population screening. BMI is commonly used for obesity classification. The traditional view holds that only individuals with elevated BMI have a higher risk of developing MASLD, but this understanding is fundamentally flawed because BMI neither reflects body fat percentage nor accurately assesses related health risks [8]. To address these limitations, researchers have developed several novel composite indices [9, 10, 11]. The logarithmic lipid accumulation product (LAP), which integrates waist circumference (WC) and triglyceride levels, has shown outstanding performance in assessing metabolic abnormalities. The body roundness index (BRI) is a superior indicator of fat distribution and visceral fat content and may be more closely associated with mortality risk than BMI, WC, or hip circumference [12]. The weight‐adjusted waist index (WWI) is less influenced by muscle mass than is BMI, while A Body Shape Index (ABSI) significantly improves mortality risk prediction by integrating WC, BMI, and height [13]. These innovative indicators offer promising new approaches for noninvasive MASLD screening across different populations. However, current evidence remains insufficient to fully validate the associations between these emerging anthropometric parameters and MASLD risk.

This study investigated the associations between novel anthropometric indices and MASLD in United States adults and evaluated their predictive value for MASLD and LF.

## 2 Materials and Methods

### 2.1 Data Source

Data were sourced from the National Health and Nutrition Examination Survey (NHANES) 2017–2020 and included demographic, physical examination, laboratory, lifestyle, and liver health parameters, including controlled attenuation parameter (CAP) and liver stiffness measurement (LSM). The study adhered to the ethical guidelines specified in NCHS Protocol Numbers 2011‐17 and 2018‐01 and followed the STROBE guidelines.

### 2.2 Diagnostic Criteria and Study Population

#### 2.2.1 Definition of MASLD

**FIGURE 1:**
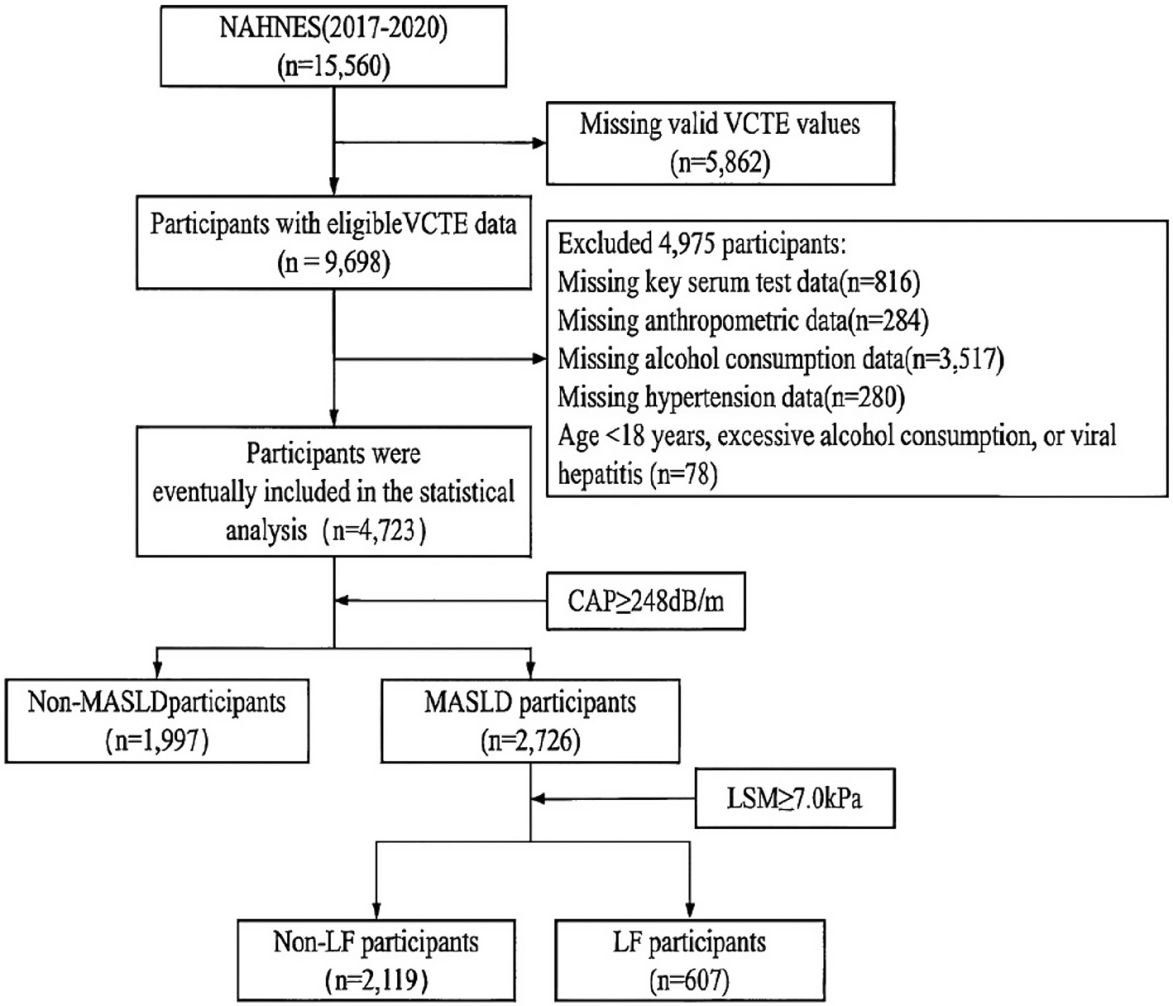
Technical roadmap.

Significant hepatic steatosis was defined as a CAP of ≥ 248 dB/m, and LF was defined as LSM of ≥ 8.2 kPa [7]. MASLD was defined as steatosis plus at least one of the following five cardiometabolic risk factors (CMRFs) [14, 15]: overweight or obesity (BMI of ≥ 25 kg/m^2^, or ≥ 23 kg/m^2^ for Asians; WC of ≥ 94 cm for men or ≥ 80 cm for women), impaired glucose regulation, hypertension (blood pressure of ≥ 130/85 mmHg or use of antihypertensive medication), hypertriglyceridemia (triglycerides of ≥ 1.70 mmol/L or use of lipid‐lowering treatment), and reduced high‐density lipoprotein cholesterol (HDL‐C) (< 1.0 mmol/L in men or < 1.3 mmol/L in women, or use of treatment). The exclusion criteria were heavy alcohol consumption, viral hepatitis, and the use of steatogenic medications. Following a multistage screening process (Figure 1), eligible participants were classified into MASLD and non‐MASLD groups. Participants with MASLD were further divided into LF and non‐LF groups according to LSM values.

#### 2.2.2 Formula for Calculating Noninvasive Predictive Scores

The following formulas were used to calculate the waist‐to‐height ratio, BRI, LAP, waist‐triglyceride index (WTI), WWI, ABSI, hepatic steatosis index (HSI), fibrosis‐4 index (FIB‐4), and aspartate aminotransferase‐to‐platelet ratio index (APRI) [16, 17, 18, 19, 20]:

$$\text{Waist}-\text{to}-\text{height ratio}=\mathrm{WC}\;\left(\mathrm{cm}\right)/\text{Height}\;\left(\mathrm{cm}\right)$$

$$\mathrm{BRI}=364.2-365.5\times \sqrt{1-{\left(\frac{\mathrm{WC}\;\left(\mathrm{cm}\right)}{2\pi }\right)}^{2}/{\left(0.5\times \text{Height}\;\left(m\right)\right)}^{2}}$$

$$\mathrm{LAP}=\left[\left(\mathrm{WC}\;\left(\mathrm{cm}\right)-65\;\left(\text{for}\;\mathrm{men}\right)\;\text{or}\;58\;\left(\text{for women}\right)\right)\times \mathrm{TG}\;\left(\text{mmol}/L\right)\right]$$

$$\mathrm{WTI}=\ln\left[\mathrm{TG}\;\left(\mathrm{mg}/\mathrm{dL}\right)\times \mathrm{WC}\;\left(\mathrm{cm}\right)/2\right]$$

$$\mathrm{WWI}=\mathrm{WC}\;\left(\mathrm{cm}\right)/\sqrt{\text{Weight}\;\left(\mathrm{kg}\right)}$$

$$\text{ABSI}=\mathrm{WC}\;\left(\mathrm{cm}\right)/\sqrt{\text{Height}\;\left(m\right)}\times \sqrt[{3}]{{\mathrm{BMI}}^{2}}$$

$$\begin{array}{c} \mathrm{HSI}=8\times \left[\mathrm{ALT}\;\left(U/L\right)/\mathrm{AST}\;\left(U/L\right)\right]\\ +\mathrm{BMI}\;\left(\mathrm{kg}/m^{2}\right)\;\left(+2,\text{if diabetes};+2,\text{if female}\right) \end{array}$$

$$\mathrm{FIB}-4=\left[\mathrm{Age}\;\left(\text{years}\right)\times \mathrm{AST}\;\left(U/L\right)\right]/\left[\mathrm{PLT}\;\left(10^{9}/L\right)\times \mathrm{ALT}\;\left(U/L\right)\right]$$

$$\text{APRI}=\left[{\text{AST(U/L)/PLT(10}}^{\text{9}}\text{/L)}\right]\times 100$$

### 2.3 Statistical Analysis

Analyses were performed using R version 4.3.1. Continuous and categorical variables were compared using *t*‐tests and chi‐square tests, respectively. Logistic regression was used to evaluate the associations of the anthropometric indices with MASLD and, among participants with MASLD, with LF. Results are reported as odds ratios (ORs) with 95% confidence intervals (CIs) across three models (unadjusted; adjusted for sex, education, marital status, race, diabetes, hypertension, and age; and further adjusted for sleep duration, alkaline phosphatase, gamma‐glutamyl transferase, total bilirubin, total cholesterol, and HDL‐C). Variance inflation factors of < 5 indicated the absence of significant multicollinearity. Risk differences across quartiles (Q1–Q4) were evaluated. Stratified analyses, restricted cubic spline curves, receiver operating characteristic (ROC) curves, and decision curve analysis (DCA) were also performed. A two‐sided *p* value of < 0.05 was considered statistically significant.

## 3 Results

### 3.1 Baseline Characteristics of Study Participants

As shown in Table 1, this study included a total of 4723 adult participants from the NHANES 2017–2020 database, of whom 2726 (57.72%) were diagnosed with MASLD. The mean age of the study population was 49.78 ± 16.39 years. Compared with the non‐MASLD group, the MASLD group had a higher proportion of men and significantly higher prevalences of diabetes and hypertension. Regarding metabolic and liver‐related indicators, participants with MASLD exhibited significantly higher levels of BMI, LSM, CAP, alanine aminotransferase, aspartate aminotransferase, gamma‐glutamyl transferase, alkaline phosphatase, blood glucose, and total cholesterol, whereas HDL‐C levels were significantly lower (all *p* < 0.05). Furthermore, all composite metabolic indices, including LAP, BRI, WTI, WWI, ABSI, and HSI, were significantly higher in the MASLD group (all *p* < 0.05).

**TABLE 1:** Weighted baseline characteristics of participants with and without MASLD as assessed by VCTE in the NHANES 2017–2020 database.

Variables	Total (*n* = 4723)	Non‐MASLD (*n* = 1997)	MASLD (*n* = 2726)	*p*
AGE (years)	46.57 ± 17.48	42.17 ± 17.95	49.78 ± 16.39	< 0.001
Gender (%)				< 0.001
Male	2468 (52.25)	941 (47.12)	1527 (56.02)	
Female	2255 (47.75)	1056 (52.88)	1199 (43.98)	
Education (%)				< 0.001
Less than high school	602 (12.75)	211 (10.57)	391 (14.34)	
More than high school	3916 (82.91)	1638 (82.02)	2278 (83.57)	
Others	205 (4.34)	148 (7.41)	57 (2.09)	
MaritaL (%)				< 0.001
Married	2656 (56.24)	982 (49.17)	1674 (61.41)	
Unmarried	903 (19.12)	356 (17.83)	547 (20.07)	
Others	1164 (24.65)	659 (33.00)	505 (18.53)	
Race, *n* (%)				< 0.001
Mexican American	602 (12.75)	179 (8.96)	423 (15.52)	
Other Hispanic	486 (10.29)	188 (9.41)	298 (10.93)	
Non‐Hispanic White	1769 (37.46)	736 (36.86)	1033 (37.89)	
Non‐Hispanic Black	1174 (24.86)	571 (28.59)	603 (22.12)	
Other races	692 (14.65)	323 (16.17)	369 (13.54)	
BMI (kg/m^2^)	29.84 ± 7.28	25.84 ± 5.07	32.76 ± 7.26	< 0.001
LSM (kPa)	5.87 ± 4.92	4.97 ± 3.64	6.53 ± 5.59	< 0.001
CAP (dB/m)	264.34 ± 62.48	205.53 ± 30.18	307.43 ± 41.32	< 0.001
ALT (U/L)	22.49 ± 16.21	18.04 ± 11.55	25.74 ± 18.23	< 0.001
ALP (IU/L)	75.65 ± 24.98	71.18 ± 25.08	78.92 ± 24.40	< 0.001
AST (U/L)	21.71 ± 11.76	20.43 ± 9.74	22.65 ± 12.96	< 0.001
GRE (mg/dL)	0.88 ± 0.32	0.88 ± 0.30	0.88 ± 0.34	0.691
GLU (mg/dL)	99.13 ± 32.51	91.62 ± 19.16	104.64 ± 38.61	< 0.001
GGT (IU/L)	31.56 ± 44.35	24.14 ± 39.58	36.99 ± 46.81	< 0.001
TBIL (mg/dL)	0.46 ± 0.28	0.48 ± 0.30	0.45 ± 0.25	< 0.001
TC (mg/dL)	186.16 ± 40.40	181.42 ± 38.54	189.63 ± 41.37	< 0.001
HDL‐C (mg/dL)	53.86 ± 15.99	58.59 ± 15.92	50.39 ± 15.14	< 0.001
BRI	5.58 ± 2.49	4.16 ± 1.73	6.63 ± 2.44	< 0.001
LAP	62.99 ± 58.73	34.70 ± 33.07	83.71 ± 64.49	< 0.001
WTI	8.64 ± 0.64	8.30 ± 0.54	8.89 ± 0.58	< 0.001
WWI	10.96 ± 0.86	10.53 ± 0.82	11.28 ± 0.74	< 0.001
ABSI	8.09 ± 0.49	7.93 ± 0.49	8.20 ± 0.45	< 0.001
HSI	37.91 ± 8.60	32.81 ± 6.02	41.65 ± 8.28	< 0.001
Sleep hours (h/day)	7.50 ± 1.56	7.59 ± 1.56	7.42 ± 1.56	< 0.001
Diabetes (%)	649 (13.74)	113 (5.66)	536 (19.66)	< 0.001
Hypertension (%)	884 (18.72)	270 (13.52)	614 (22.52)	< 0.001

*Note:* Continuous variables are shown as mean (SE) and their *p* value was calculated by linear regression model. Categorical values are shown as % (SE) and its *p* value was calculated by chi‐square test.

The weighted baseline characteristics of participants with and without LF in the MASLD group are summarized in Table S1.

### 3.2 Associations Between Five Novel Anthropometric Indices and the Risk of MASLD and LF in MASLD

To evaluate the effects of various obesity‐related indices, logistic regression analyses were performed using these indices as both categorical variables (quartiles) and continuous variables (per one standard deviation increase). In the continuous analysis, all indices showed significant positive associations with MASLD. When analyzed by quartiles, a consistent dose–response trend was observed across all indices, with progressively higher ORs in ascending quartiles compared with the reference Q1 group, as shown in Table 2A.

**TABLE 2A:** Binary logistic regression analysis of the associations between five novel anthropometric indices and MASLD.

Variables	Model 1		Model 2		Model 3	
	OR (95% CI)	*p*	OR (95% CI)	*p*	OR (95% CI)	*p*
Baseline WTI (per SD increase)	6.73 (5.90, 7.67)	< 0.001	5.95 (5.20, 6.81)	< 0.001	5.31 (4.47, 6.31)	< 0.001
WTI group						
Q1	1.00 (Reference)		1.00 (Reference)		1.00 (Reference)	
Q2	3.07 (2.58, 3.66)	< 0.001	2.60 (2.17, 3.12)	< 0.001	2.34 (1.93, 2.83)	< 0.001
Q3	7.52 (6.27, 9.03)	< 0.001	5.94 (4.91, 7.18)	< 0.001	4.79 (3.88, 5.93)	< 0.001
Q4	16.93 (13.77, 20.82)	< 0.001	12.61 (10.16, 15.65)	< 0.001	8.96 (6.87, 11.69)	< 0.001
Baseline WWI (per SD increase)	3.48 (3.18, 3.80)	< 0.001	4.12 (3.70, 4.60)	< 0.001	3.31 (2.95, 3.71)	< 0.001
WWI group						
Q1	1.00 (Reference)		1.00 (Reference)		1.00 (Reference)	
Q2	3.71 (3.11, 4.42)	< 0.001	3.73 (3.09, 4.50)	< 0.001	2.95 (2.43, 3.58)	< 0.001
Q3	7.44 (6.20, 8.92)	< 0.001	7.47 (6.08, 9.19)	< 0.001	5.41 (4.36, 6.70)	< 0.001
Q4	12.98 (10.67, 15.80)	< 0.001	14.89 (11.74, 18.89)	< 0.001	10.03 (7.83, 12.85)	< 0.001
Baseline ABSI (per SD increase)	3.45 (3.02, 3.94)	< 0.001	2.61 (2.23, 3.06)	< 0.001	2.13 (1.80, 2.51)	< 0.001
ABSI group						
Q1	1.00 (Reference)		1.00 (Reference)		1.00 (Reference)	
Q2	2.25 (1.91, 2.66)	< 0.001	1.82 (1.53, 2.16)	< 0.001	1.58 (1.32, 1.90)	< 0.001
Q3	3.70 (3.12, 4.39)	< 0.001	2.62 (2.17, 3.15)	< 0.001	2.21 (1.82, 2.69)	< 0.001
Q4	4.60 (3.87, 5.48)	< 0.001	2.86 (2.32, 3.52)	< 0.001	2.35 (1.88, 2.92)	< 0.001
Baseline BRI (per SD increase)	1.95 (1.87, 2.04)	< 0.001	1.98 (1.89, 2.07)	< 0.001	1.86 (1.77, 1.95)	< 0.001
BRI group						
Q1	1.00 (Reference)		1.00 (Reference)		1.00 (Reference)	
Q2	5.66 (4.67, 6.86)	< 0.001	5.05 (4.13, 6.18)	< 0.001	4.28 (3.47, 5.27)	< 0.001
Q3	14.02 (11.47, 17.14)	< 0.001	12.72 (10.25, 15.78)	< 0.001	10.03 (8.00, 12.56)	< 0.001
Q4	37.64 (29.79, 47.56)	< 0.001	40.75 (31.60, 52.56)	< 0.001	30.87 (23.62, 40.36)	< 0.001
Baseline LAP (per SD increase)	1.04 (1.03, 1.04)	< 0.001	1.03 (1.03, 1.04)	< 0.001	1.03 (1.03, 1.04)	< 0.001
LAP group						
Q1	1.00 (Reference)		1.00 (Reference)		1.00 (Reference)	
Q2	4.83 (4.00, 5.84)	< 0.001	4.17 (3.43, 5.08)	< 0.001	4.28 (3.48, 5.25)	< 0.001
Q3	13.60 (11.14, 16.59)	< 0.001	11.38 (9.26, 14.00)	< 0.001	11.68 (9.24, 14.77)	< 0.001
Q4	38.83 (30.64, 49.20)	< 0.001	30.14 (23.58, 38.52)	< 0.001	31.12 (23.19, 41.75)	< 0.001

To further evaluate participants with MASLD, both continuous and quartile‐based analyses indicated that higher levels of WTI, WWI, BRI, and LAP were significantly associated with an increased risk of LF. When comparing the highest and lowest quartiles, BRI demonstrated the strongest association among all indices. By contrast, although ORs for ABSI increased from Q1 to Q4 in Model 1, the association weakened and became nonsignificant after adjustment in Models 2 and 3, as shown in Table 2B.

**TABLE 2B:** Binary logistic regression analysis of the associations between five novel anthropometric indices and LF in MASLD.

Variables	Model 1		Model 2		Model 3	
	OR (95% CI)	*p*	OR (95% CI)	*p*	OR (95% CI)	*p*
Baseline WTI (per SD increase)	1.95 (1.67, 2.28)	< 0.001	2.01 (1.70, 2.37)	< 0.001	2.01 (1.61, 2.52)	< 0.001
WTI group						
Q1	1.00 (Reference)		1.00 (Reference)		1.00 (Reference)	
Q2	2.27 (1.43, 3.60)	< 0.001	2.12 (1.33, 3.39)	0.002	2.02 (1.25, 3.26)	0.004
Q3	3.16 (2.04, 4.91)	< 0.001	2.83 (1.80, 4.43)	< 0.001	2.73 (1.70, 4.38)	< 0.001
Q4	4.27 (2.77, 6.58)	< 0.001	3.57 (2.29, 5.58)	< 0.001	3.35 (2.03, 5.52)	< 0.001
Baseline WWI (per SD increase)	2.00 (1.76, 2.27)	< 0.001	2.30 (1.98, 2.67)	< 0.001	10 (1.80, 2.46)	< 0.001
WWI group						
Q1	1.00 (Reference)		1.00 (Reference)		1.00 (Reference)	
Q2	1.07 (0.69, 1.67)	0.746	1.05 (0.67, 1.64)	0.842	0.99 (0.63,1.56)	0.965
Q3	2.54 (1.70, 3.79)	< 0.001	2.36 (1.56, 3.59)	< 0.001	2.15 (1.40, 3.29)	< 0.001
Q4	3.67 (2.48, 5.44)	< 0.001	3.57 (2.33, 5.48)	< 0.001	3.12 (2.01, 4.85)	< 0.001
Baseline ABSI (per SD increase)	1.56 (1.28, 1.91)	< 0.001	1.31 (1.03, 1.66)	< 0.001	1.14 (0.88, 1.46)	< 0.058
ABSI group						
Q1	1.00 (Reference)		1.00 (Reference)		1.00 (Reference)	
Q2	0.97 (0.71, 1.34)	0.864	0.85 (0.61, 1.19)	0.349	0.84 (0.60, 1.17)	0.299
Q3	1.56 (1.16, 2.10)	0.003	1.24 (0.90, 1.71)	0.182	1.21 (0.88, 1.67)	0.248
Q4	1.60 (1.19, 2.15)	0.002	1.11 (0.79, 1.56)	0.557	1.00 (0.70, 1.41)	0.978
Baseline BRI (per SD increase)	1.36 (1.31, 1.42)	< 0.001	1.44 (1.38, 1.50)	< 0.001	1.43 (1.36, 1.49)	< 0.001
BRI group						
Q1	1.00 (Reference)		1.00 (Reference)		1.00 (Reference)	
Q2	1.51 (0.83, 2.75)	0.182	1.30 (0.71, 2.39)	0.394	1.36 (0.72, 2.57)	0.341
Q3	2.64 (1.49, 4.68)	< 0.001	2.16 (1.21, 3.86)	0.009	2.18 (1.18, 4.03)	0.013
Q4	7.46 (4.27, 13.04)	< 0.001	6.65 (3.76, 11.75)	< 0.001	6.80 (3.71, 12.47)	< 0.001
Baseline LAP (per SD increase)	1.21 (1.01, 1.30)	< 0.001	1.09 (1.01, 1.21)	< 0.001	1.02 (1.01, 1.08)	< 0.001
LAP group						
Q1	1.00 (Reference)		1.00 (Reference)		1.00 (Reference)	
Q2	1.45 (0.82, 2.57)	0.204	1.27 (0.71, 2.25)	0.424	1.37 (0.76, 2.48)	0.298
Q3	3.17 (1.86, 5.42)	< 0.001	2.71 (1.58, 4.66)	< 0.001	3.17 (1.79, 5.63)	< 0.001
Q4	5.67 (3.35, 9.59)	< 0.001	4.44 (2.60, 7.58)	< 0.001	5.45 (3.02, 9.82)	< 0.001

*Note:* Model 1: Crude.

Model 2: Adjusted: Sex, education, marital status, race, diabetes status, hypertension status, age, sleep duration.

Model 3: Adjusted: Sex, education, marital status, race, diabetes status, hypertension status, age, sleep duration, ALP, GGT, TBIL, TC, and HDL‐C.

Abbreviations: CI, confidence interval; OR, odds ratio.

Table 3A shows significant interactions between several obesity‐related indices and key demographic and metabolic factors. In the sex‐stratified analysis, BRI, ABSI, and WTI showed statistically significant interactions (all *p* for interaction < 0.05). WTI had the strongest association with MASLD in women (OR = 6.27), whereas ABSI (OR = 3.43) and BRI (OR = 2.15) showed stronger associations in men. In the age‐stratified analysis, WWI, ABSI, BRI, and LAP showed significant interactions (all *p* for interaction < 0.05). WWI, ABSI, and BRI exhibited the strongest associations in the 18‐ to 45‐year age group, with ORs of 4.04, 2.58, and 1.94, respectively. In the BMI‐stratified analysis, WWI, BRI, and LAP showed significant interactions (all *p* for interaction < 0.05). The association between WTI and MASLD strengthened with increasing BMI and was greatest in participants with a BMI of > 35 kg/m^2^ (OR = 4.64), whereas the associations of WWI, ABSI, and BRI gradually weakened. In the race‐stratified analysis, LAP showed the strongest association in non‐Hispanic Black participants (OR = 1.05). In the hypertension‐stratified analysis, the association of WWI was stronger in participants without hypertension than in those with hypertension, whereas differences for the other indices were relatively small. In the diabetes‐stratified analysis, WWI, ABSI, BRI, and LAP showed stronger associations in participants without diabetes (all *p* for interaction < 0.05).

**TABLE 3A:** Weighted stratified associations between five novel anthropometric indicators‐related indices and MASLD by age, sex, BMI, race, hypertension, and diabetes.

Subgroup	WTI		WWI		ABSI		BRI		LAP	
	OR (95% CI)	*p* for interaction	OR (95% CI)	*p* for interaction	OR (95% CI)	*p* for interaction	OR (95% CI)	*p* for interaction	OR (95% CI)	*p* for interaction
Gender		0.01		0.06		0.006		< 0.001		0.381
Female	6.27 (4.81, 8.17)		2.61 (2.25, 3.03)		1.54 (1.24, 1.9)		1.62 (1.53, 1.72)		1.03 (1.03, 1.03)	
Male	4.26 (3.4,5.34)		4.27 (3.55, 5.12)		3.43 (2.61, 4.52)		2.15 (1.98, 2.33)		1.03 (1.03, 1.04)	
Age (years)		0.185		< 0.001		< 0.001		0.001		0.007
18–45	4.33 (3.42, 5.49)		4.04 (3.39, 4.82)		2.58 (2.01, 3.31)		1.94 (1.8,2.08)		1.03 (1.03, 1.04)	
45–60	5.09 (3.57, 7.28)		2.95 (2.33, 3.72)		1.61 (1.16, 2.24)		1.82 (1.64,2.02)		1.03 (1.02, 1.03)	
≥ 60	5.64 (3.94, 8.08)		2.5 (2.01,3.1)		1.97 (1.44, 2.7)		1.63 (1.49, 1.78)		1.03 (1.02, 1.04)	
BMI (kg/m^2^)		0.177		0.043		0.142		< 0.001		0.005
< 25	2.74 (1.92, 3.92)		2.65 (1.80, 3.91)		2.58 (1.75, 3.82)		2.50 (1.98, 3.15)		1.03 (1.02, 1.04)	
25–35	3.08 (2.44, 3.88)		2.60 (2.03, 3.31)		2.47 (1.94, 3.16)		1.77 (1.61, 1.94)		1.02 (1.01, 0.02)	
> 35	4.64 (2.55, 8.42)		1.95 (1.25, 3.05)		1.93 (1.24, 3.03)		1.48 (1.29,1.69)		1.02 (1.01, 1.03)	
Race		0.564		0.658		0.811		0.716		0.018
Mexican American	3.57 (2.04, 6.24)		2.65 (1.83, 3.83)		1.85 (1.05, 3.24)		1.81 (1.53, 2.14)		1.02 (1.01, 1.03)	
Other Hispanic	4.42 (2.55, 7.66)		3.06 (2.09, 4.47)		1.87 (1.08, 3.24)		1.72 (1.48, 2.01)		1.02 (1.02, 1.03)	
Non‐Hispanic White	4.73 (3.55, 6.3)		3.27 (2.7, 3.96)		1.82 (1.38, 2.39)		1.89 (1.74, 2.05)		1.03 (1.02, 1.03)	
Non‐Hispanic Black	7.07 (4.96, 10.07)		3.25 (2.63,4.02)		2.09 (1.53,2.86)		1.79 (1.65, 1.95)		1.05 (1.04, 1.05)	
Other race	4.51 (2.94, 6.91)		3.51 (2.54, 4.85)		2.68 (1.68, 4.29)		2.08 (1.77, 2.44)		1.03 (1.02, 1.04)	
Hypertension		0.93		0.029		0.058		0.127		0.24
No	4.82 (4, 5.82)		3.26 (2.87, 3.71)		2.06 (1.71, 2.47)		1.83 (1.73, 1.93)		1.03 (1.03, 1.03)	
Yes	5.64 (3.7, 8.6)		2.95 (2.27, 3.83)		2.00 (1.38, 2.9)		1.75 (1.57, 1.95)		1.03 (1.02, 1.04)	
Diabetes		0.409		0.021				0.013		< 0.001
No	4.93 (4.13, 5.9)		3.35 (2.96, 3.78)		2.15 (1.81, 2.56)	0.004	1.86 (1.76, 1.96)		1.03 (1.03, 1.04)	
Yes	4.94 (2.82, 8.66)		2.14 (1.48, 3.08)		1.22 (0.7, 2.13)		1.53 (1.34, 1.75)		1.02 (1.01, 1.03)	

Among participants with MASLD, age showed significant interactions with WWI and ABSI in relation to LF risk (both *p* for interaction < 0.05), with both indices showing the strongest associations in the 18‐ to 45‐year age group (OR = 2.68 and 1.87, respectively) (Table 3B). Hypertension showed significant interactions with WWI and BRI, with both indices demonstrating stronger associations in participants without hypertension (OR = 2.16 and 1.45, respectively). Diabetes showed a significant interaction with ABSI (OR = 1.14). No significant interactions were observed between sex, BMI, or race and any of the indices (all *p* for interaction > 0.05). Detailed weighted stratified associations between the five novel anthropometric indices and LF in MASLD according to age, sex, BMI, race, hypertension, and diabetes are presented in Table S3.

**TABLE 3B:** Weighted stratified associations between five novel anthropometric indicators‐related indices and LF in MASLD by age, sex, BMI, race, hypertension, and diabetes.

Subgroup	WTI		WWI		ABSI		BRI		LAP	
	OR (95% CI)	*p* for interaction	OR (95% CI)	*p* for interaction	OR (95% CI)	*p* for interaction	OR (95% CI)	*p* for interaction	OR (95% CI)	*p* for interaction
Gender		0.103		0.265		0.117		0.058		0.112
Female	2.13 (1.46, 3.13)		1.7 (1.36, 2.13)		0.79 (0.57, 1.11)		1.37 (1.29, 1.46)		1.01 (1, 1.01)	
Male	1.75 (1.32, 2.32)		2.41 (1.92, 3.03)		1.62 (1.11, 2.37)		1.48 (1.38, 1.59)		1.01 (1, 1.01)	
Age (years)		0.581		0.049		0.006		0.862		0.479
18–45	1.89 (1.29, 2.75)		2.68 (2.01, 3.57)		1.87 (1.16, 3.02)		1.46 (1.35, 1.58)		1.01 (1, 1.01)	
45–60	1.83 (1.22, 2.76)		1.82 (1.38, 2.39)		0.82 (0.53, 1.26)		1.41 (1.3, 1.53)		1.01 (1, 1.01)	
≥ 60	1.78 (1.19, 2.66)		1.82 (1.38, 2.42)		0.97 (0.64, 1.47)		1.39 (1.27, 1.51)		1.01 (1, 1.01)	
BMI (kg/m^2^)		0.658		0.334		0.383		0.471		0.665
< 25	4.68 (1.62, 13.54)		1.15 (0.48, 2.73)		1.37 (0.39, 4.82)		1.35 (0.68, 2.65)		1.02 (1.00, 1.04)	
25–35	1.57 (1.11, 2.21)		1.38 (1.06, 1.80)		1.45 (0.97, 2.18)		1.29 (1.13, 1.48)		1.00 (1.00, 1.01)	
> 35	1.27 (0.88, 1.85)		1.52 (1.20, 1.93)		1.22 (0.85, 1.75)		1.32 (1.22, 1.42)		1.00 (1.00, 1.00)	
Race		0.246		0.466		0.761		0.449		0.073
Mexican American	1.91 (1.03, 3.55)		2.23 (1.48, 3.38)		0.99 (0.42, 2.38)		1.49 (1.3, 1.71)		1 (1, 1.01)	
Other Hispanic	2.39 (1.15, 4.94)		3.4 (1.98, 5.83)		2.38 (1.04, 5.44)		1.62 (1.37, 1.93)		1.01 (1, 1.02)	
Non‐Hispanic White	1.83 (1.26, 2.65)		1.82 (1.41, 2.36)		1.18 (0.71, 1.98)		1.39 (1.29, 1.5)		1.01 (1, 1.01)	
Non‐Hispanic Black	2.21 (1.38, 3.55)		1.91 (1.39, 2.63)		0.83 (0.55, 1.25)		1.35 (1.24, 1.47)		1.01 (1.01, 1.02)	
Other race	1.91 (1.03, 3.55)		1.94 (1.11, 3.38)		1.05 (0.55, 2.02)		1.54 (1.3, 1.82)		1.01 (1, 1.01)	
Hypertension		0.118		0.009		0.169		0.037		0.315
No	2.05 (1.58, 2.66)		2.16 (1.8, 2.6)		1.07 (0.8, 1.43)		1.45 (1.38, 1.54)		1.01 (1, 1.01)	
Yes	1.26 (0.8, 1.98)		1.71 (1.24, 2.37)		1.09 (0.66, 1.79)		1.32 (1.21, 1.44)		1 (1, 1.01)	
Diabetes		0.39		0.688		0.03		0.073		0.837
No	1.79 (1.38, 2.33)		1.94 (1.62, 2.33)		1.14 (0.85, 1.52)		1.38 (1.31, 1.46)		1.01 (1, 1.01)	
Yes	2.09 (1.35, 3.23)		2.21 (1.58, 3.09)		0.88 (0.54, 1.45)		1.49 (1.35, 1.65)		1.01 (1, 1.01)	

*Note:* Adjust for sex, education, marital status, race, diabetes status, hypertension status, age, sleep duration, ALP, GGT, TBIL, TC, and HDL‐C. In the subgroup analysis, models were not adjusted for their own stratification variable.

**FIGURE 2:**
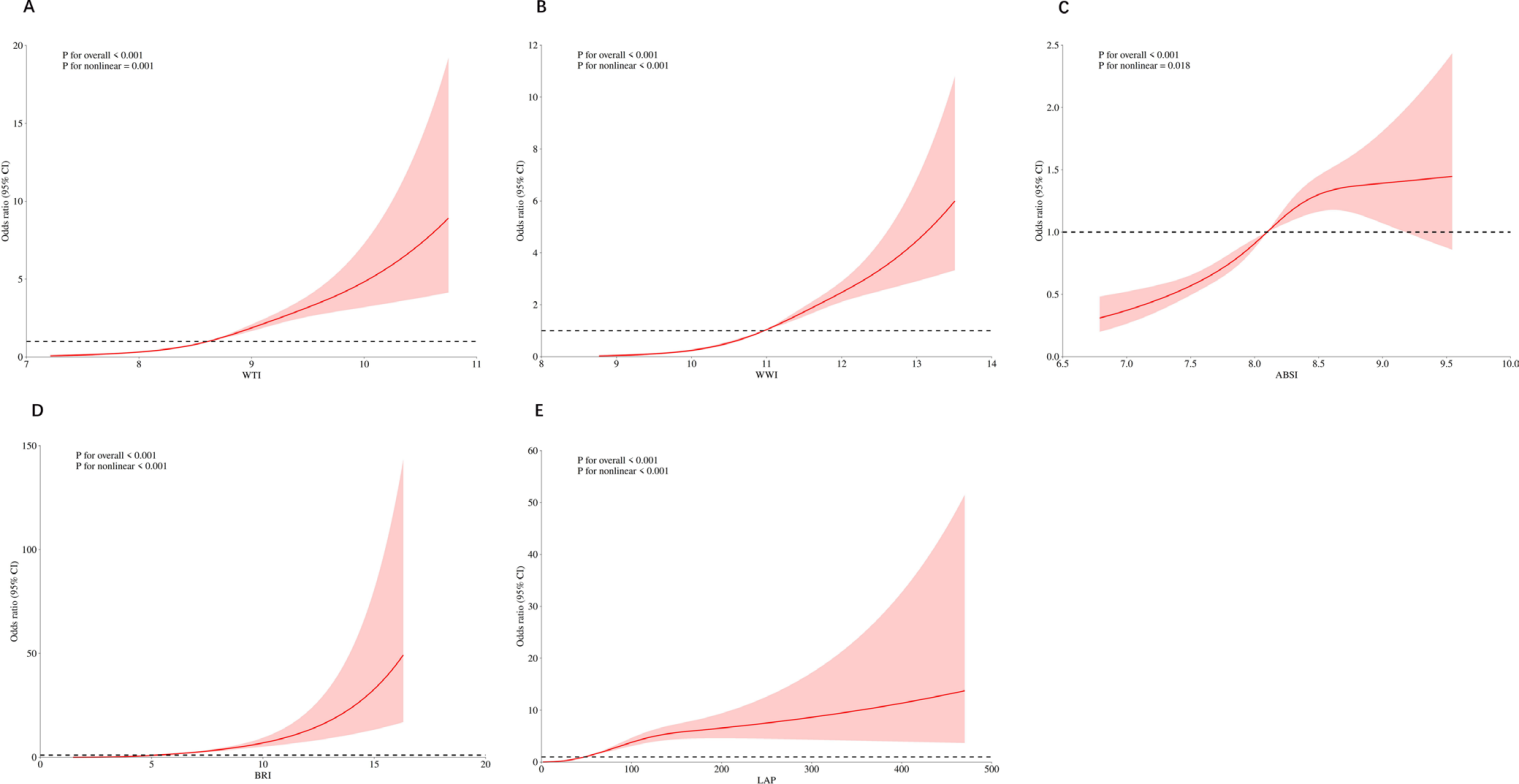
The associations between MASLD risk and (A) WTI, (B) WII, (C) ABSI, (D) BRI, and (E) LAP, represented by RCS with 95% confidence intervals.

Additionally, Figure 2 presents restricted cubic spline analyses examining the associations between five anthropometric indices (WTI, WWI, ABSI, BRI, and LAP) and MASLD. Significant nonlinear associations were observed for all indices (all *p* < 0.05), using adjustment strategies consistent with the fully adjusted model. Threshold effect analysis identified a significant nonlinear relationship between BRI and MASLD risk, with an inflection point at 3.6 (results for the remaining indices are provided in Table S2). Below this threshold, BRI was associated with a substantially lower risk of MASLD (OR = 0.27, *p* < 0.001), corresponding to a 73% risk reduction. Above the threshold, BRI was positively associated with MASLD risk (OR = 2.44, *p* < 0.001), corresponding to a 144% increase in risk. Among all indices evaluated, BRI demonstrated the most pronounced threshold effect.

**FIGURE 3:**
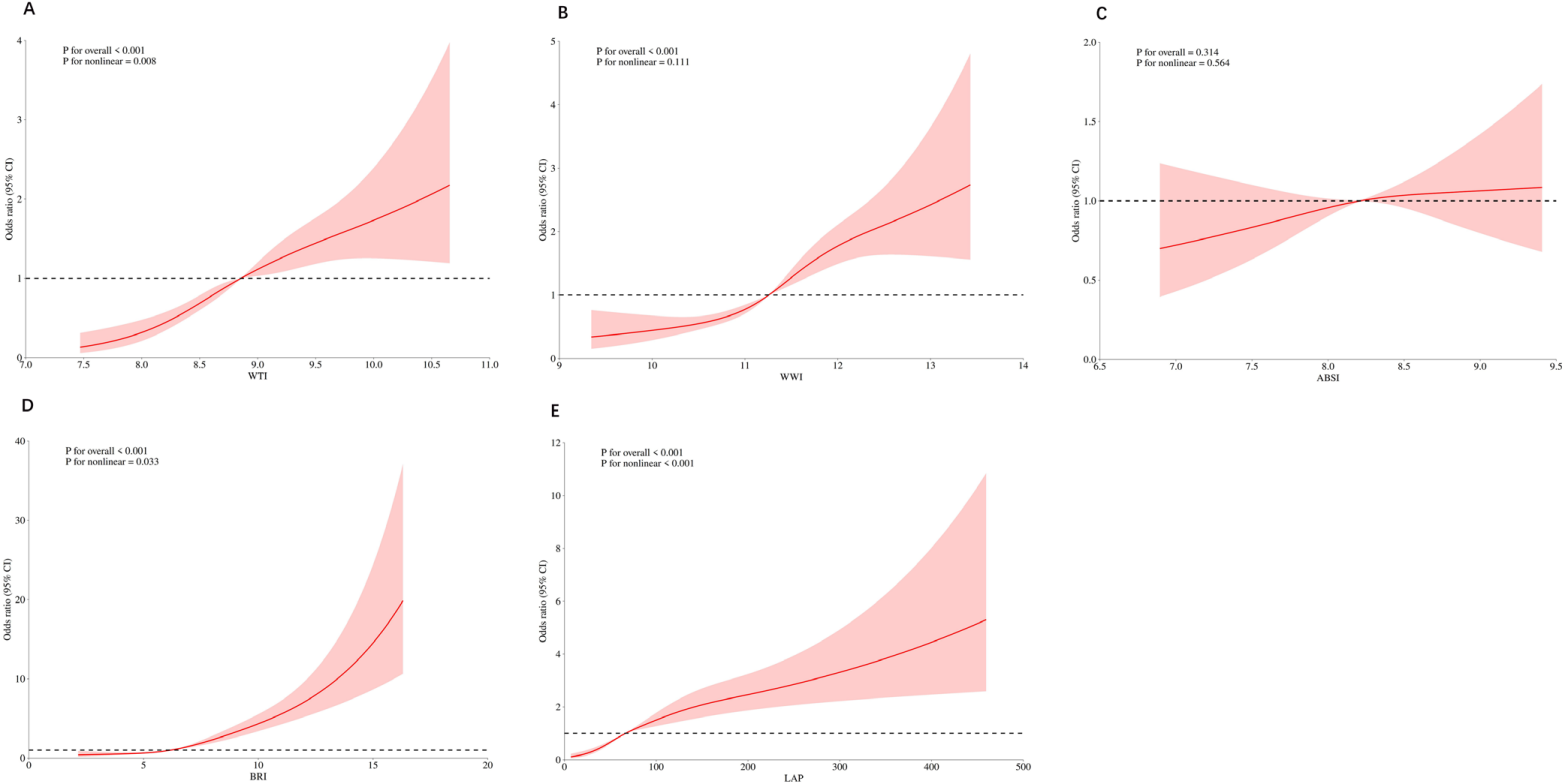
The associations between LF risk in MASLD and (A) WTI, (B) WII, (C) ABSI, (D) BRI, and (E) LAP, represented by RSC with 95% confidence intervals.

Further analysis of the relationship between LF and the anthropometric indices in participants with MASLD revealed a significant linear association between WWI and LF, whereas the association between ABSI and LF was not statistically significant, as shown in Figure 3.

### 3.3 Comparative Predictive Performance of BRI Versus Other Anthropometric Indices for MASLD and LF in MASLD: ROC Curve Analysis

HSI and FIB‐4 are widely used noninvasive models in clinical practice for assessing MASLD and LF, respectively. BMI is also commonly used for clinical screening, while APRI represents another important noninvasive index for LF assessment. To evaluate the performance of BRI and other anthropometric indices in identifying MASLD, ROC curve analysis was performed to assess the sensitivity and specificity of BMI, WTI, WWI, ABSI, BRI, LAP, and HSI for predicting MASLD. Using the same methodology, the performance of BMI, LAP, BRI, WTI, WWI, APRI, and FIB‐4 in identifying LF among participants with MASLD was also compared.

**FIGURE 4:**
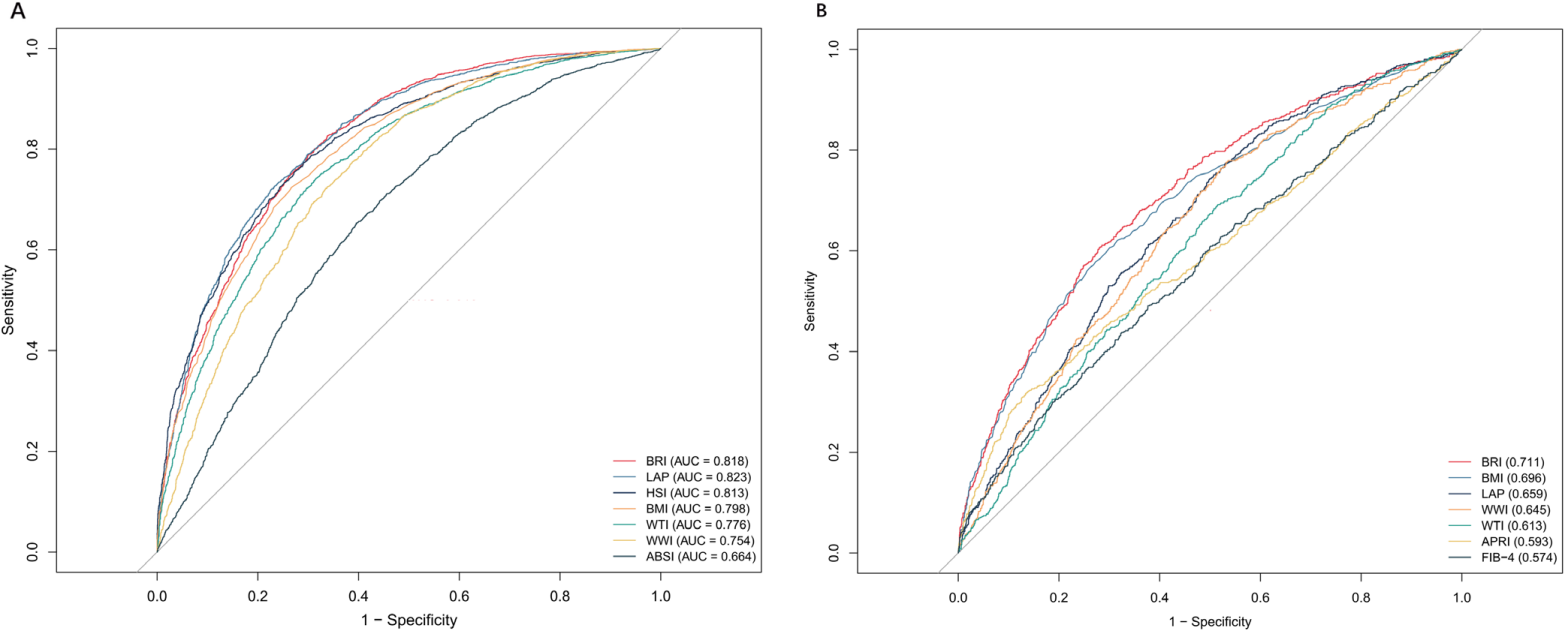
ROC curves of different anthropometric indicators for diagnosing MASLD and LF in MASLD. (A) ROC curves of anthropometric indicators including BRI, LAP, HSI, BMI, WTI, WWI, and ABSI for diagnosing MASLD; (B) ROC curves of indicators including BRI, BMI, LAP, WWI, WTI, APRI, and FIB‐4 for diagnosing MASLD with liver fibrosis.

As shown in Figure 4 and Tables 4A and 4B, both BRI and LAP demonstrated good predictive performance for MASLD, with the highest AUC reaching 0.82. This performance was superior to that of HSI (AUC = 0.81), BMI (AUC = 0.79), and the other anthropometric indices (all *p* < 0.001). For predicting LF among participants with MASLD, BRI showed relatively strong predictive ability (AUC = 0.71) and performed significantly better than BMI (AUC = 0.69), APRI (AUC = 0.59), and FIB‐4 (AUC = 0.57).

**TABLE 4A:** Performance evaluation of LAP, BRI, WTI, WWI, and ABSI for predicting MASLD.

Variables	AUC (95% CI)	SEN (95% CI)	SPE (95% CI)	PPV (95% CI)	NPV (95% CI)	Cut off value
WTI	0.78 (0.76, 0.79)	0.74 (0.73, 0.76)	0.68 (0.66, 0.7)	0.76 (0.75, 0.78)	0.66 (0.64, 0.68)	8.51
WWI	0.75 (0.74, 0.77)	0.72 (0.7, 0.74)	0.67 (0.65, 0.69)	0.75 (0.73, 0.76)	0.64 (0.62, 0.66)	10.85
ABSI	0.66 (0.65, 0.68)	0.65 (0.63, 0.67)	0.60 (0.58, 0.63)	0.69 (0.67, 0.71)	0.56 (0.54, 0.58)	8.04
BRI	0.82 (0.81, 0.83)	0.79 (0.77, 0.8)	0.70 (0.68, 0.72)	0.78 (0.77, 0.8)	0.71 (0.69, 0.73)	4.75
LAP	0.82 (0.81, 0.84)	0.72 (0.7, 0.74)	0.77 (0.75, 0.79)	0.81 (0.8, 0.83)	0.67 (0.65, 0.69)	46.43
BMI	0.79 (0.78, 0.81)	0.72 (0.70, 0.74)	0.73 (0.72, 0.76)	0.79 (0.77, 0.81)	0.66 (0.64, 0.68)	28.15
HSI	0.81 (0.8, 0.83)	0.77 (0.76, 0.79)	0.71 (0.69, 0.73)	0.78 (0.77, 0.8)	0.70 (0.68, 0.72)	35.46

**TABLE 4B:** Performance evaluation of LAP, BRI, WTI, and WWI for predicting LF in MASLD.

Variables	AUC (95% CI)	SEN (95% CI)	SPE (95% CI)	PPV (95% CI)	NPV (95% CI)	Cut off value
WTI	0.61 (0.59, 0.64)	0.69 (0.65, 0.73)	0.49 (0.46, 0.51)	0.28 (0.26, 0.30)	0.85 (0.83, 0.87)	8.78
WWI	0.65 (0.62, 0.67)	0.78 (0.74, 0.81)	0.47 (0.45, 0.49)	0.30 (0.27, 0.32)	0.88 (0.86, 0.90)	11.09
BRI	0.71 (0.60, 0.73)	0.61 (0.57, 0.64)	0.72 (0.70, 0.74)	0.38 (0.3, 0.41)	0.87 (0.85, 0.88)	7.11
LAP	0.66 (0.64, 0.68)	0.75 (0.72,0.78)	0.49 (0.47, 0.51)	0.30 (0.28, 0.32)	0.87 (0.85, 0.89)	60.56
BMI	0.69 (0.67, 0.72)	0.62 (0.58, 0.66)	0.68 (0.66, 0.70)	0.36 (0.33, 0.39)	0.86 (0.84, 0.88)	33.45
APRI	0.59 (0.57, 0.62)	0.32 (0.28, 0.36)	0.87 (0.85, 0.88)	0.4 (0.36, 0.45)	0.81 (0.8, 0.83)	0.32
FIB‐4	0.57 (0.55, 0.60)	0.49 (0.45, 0.53)	0.62 (0.60, 0.64)	0.27 (0.25, 0.30)	0.81 (0.79, 0.83)	1.02

**FIGURE 5:**
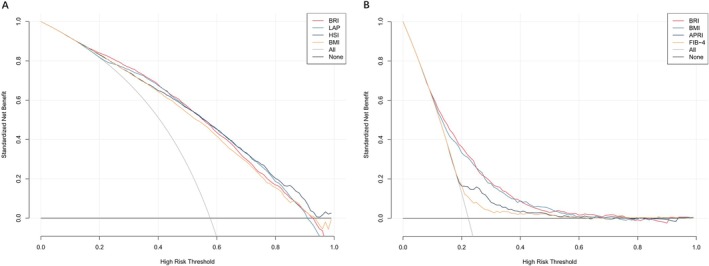
DCA curves of different anthropometric indicators for diagnosing MASLD and LF in MASLD. (A) Standardized net benefit curves of anthropometric indicators including BRI, LAP, HSI, and BMI. (B) Standardized net benefit curves of indicators including BRI, BMI, APRI, and FIB‐4.

DCA was performed to evaluate the clinical utility of BRI, LAP, BMI, and HSI for MASLD screening, as well as BRI, BMI, APRI, and FIB‐4 for predicting LF in participants with MASLD. As shown in Figure 5A, all four screening indices demonstrated positive standardized net benefit across a wide range of clinically meaningful risk thresholds (0.0–0.8), outperforming both the treat‐all and treat‐none strategies. BRI, LAP, and HSI consistently showed greater net benefit than BMI. For LF prediction (Figure 5B), BRI and BMI demonstrated positive net benefit across threshold probabilities ranging from 0 to 0.8, whereas APRI and FIB‐4 provided benefit only at very low thresholds (< 0.2), offering limited clinical value in most practical settings. Notably, BRI achieved the highest net benefit within the commonly used threshold range of 0.1–0.3, highlighting its superior clinical utility for LF risk stratification in participants with MASLD compared with traditional indices.

As shown in Table S4, within the non‐MASLD population, all five anthropometric indices were significantly higher among individuals with CMRFs than among those without CMRFs (all *p* < 0.001), confirming the potential confounding effect of CMRFs. To address this issue, a sensitivity analysis was performed by restricting the control group to non‐MASLD individuals with at least one CMRF. The results are presented in Figure S1. Following this restriction, BRI continued to demonstrate good diagnostic performance for MASLD (AUC = 0.76, 95% CI: 0.74–0.77), comparable to that observed in the primary analysis (AUC = 0.82). Furthermore, DCA showed that BRI, LAP, HSI, and BMI all provided positive net benefit across a range of threshold probabilities in this sensitivity analysis, with BRI and LAP demonstrating slightly greater net benefit at lower threshold probabilities (Figure S2).

## 4 Discussion

This study of 4723 participants, including 2726 individuals with MASLD (of whom 607 had LF), is the largest population‐based analysis to date examining the associations of five anthropometric indices—BRI, LAP, WTI, WWI, and ABSI—with MASLD. The findings indicate that nearly half of United States adults have MASLD and that all five indices are independently associated with MASLD risk. Among these indices, BRI and LAP demonstrated the strongest predictive performance for MASLD, while BRI showed the best performance for predicting LF risk among individuals with MASLD.

MASLD is a chronic condition that develops over many years, making regular screening and timely diagnosis crucial for preventing severe complications such as hepatocellular carcinoma and cirrhosis. However, diagnosis and screening remain challenging because of their cost and limited accessibility. Current research primarily relies on BMI as a key indicator for predicting MASLD. However, BMI is limited by its inability to distinguish whether excess body weight is attributable to muscle mass or adipose tissue, and it does not reflect the fat distribution [21, 22, 23]. BRI, a superior indicator of fat distribution and visceral adiposity [24], showed a significant association with MASLD (OR = 1.86), with individuals in the highest quartile (Q4) having a substantially higher risk than those in the lowest quartile (Q1). A large community‐based study from northern Iran involving 4872 participants similarly reported a strong association between BRI and ultrasound‐diagnosed MASLD (OR = 5.484 for men and OR = 3.482 for women) [25]. Furthermore, BRI demonstrated a larger area under the curve (AUC = 0.82) and higher sensitivity (79%) than HSI (AUC = 0.81; sensitivity, 77%) and BMI (AUC = 0.79; sensitivity, 72%), findings consistent with those reported by Jiang and Zhao [26, 27]. BRI also outperformed APRI (AUC = 0.59) and FIB‐4 (AUC = 0.57) in predicting LF among individuals with MASLD (AUC = 0.71). LAP integrates WC and triglyceride levels, reflecting visceral obesity and insulin resistance in MASLD [28]. Its predictive performance for MASLD (AUC = 0.82) was comparable to that of BRI; however, its association with MASLD as a continuous variable was weaker (OR = 1.03). In addition, a meta‐analysis including 16 studies reported that some studies found this association to be nonsignificant [29]. WTI showed higher ORs (OR = 5.31 for MASLD and OR = 2.01 for LF) but lower AUC values (0.78 for MASLD and 0.61 for LF). ABSI, a unique indicator for assessing central obesity and its associated health risks [30], showed the poorest predictive performance for MASLD (AUC = 0.66) and no significant association with LF.

Stratified analyses revealed sex‐specific differences. Men are more prone to visceral fat accumulation, making central obesity indices such as ABSI and BRI more predictive in men, whereas women predominantly exhibit subcutaneous fat distribution, making WTI, which comprehensively assesses overall and gluteal fat, more relevant in women [31, 32]. Age also influenced these associations, with central obesity indices (WWI, ABSI, and BRI) showing greater sensitivity in younger adults, whereas composite indices such as WTI appeared to have greater clinical relevance in older adults [33]. BMI further modified these associations: the predictive strength of WTI increased with increasing BMI, whereas that of WWI, ABSI, and BRI decreased. This pattern may reflect the predominance of systemic metabolic disturbances in severe obesity, which could attenuate the predictive value of central obesity indices. Furthermore, LAP showed stronger associations in non‐Hispanic Black individuals and in participants without diabetes, reflecting metabolic heterogeneity in visceral obesity and insulin resistance across different populations [34]. Hypertension and diabetes weakened the associations of WWI and other central obesity indices with MASLD, suggesting that in individuals with established metabolic disease, the pathogenesis of MASLD may shift from body shape‐related factors toward systemic metabolic dysfunction [33].

Among participants with MASLD and LF, no significant interactions were observed between sex, BMI, or race and any anthropometric index. Instead, age and metabolic disease status appeared to play more important roles. Notably, WWI and ABSI demonstrated the strongest predictive ability in younger adults, likely because early‐onset MASLD is more metabolically driven and progresses more rapidly; consequently, indices reflecting central obesity and body fat distribution may have greater value in this population [35]. Furthermore, diabetes specifically attenuated the predictive value of ABSI for LF, further emphasizing the importance of considering individual characteristics when selecting anthropometric indices for MASLD risk stratification.

Beyond their role in MASLD risk assessment, these novel anthropometric indices may have broader clinical utility. Cardiovascular disease is the leading cause of death among patients with MASLD [36], and several studies have shown that BRI has considerable potential for predicting cardiovascular disease risk [37, 38]. Therefore, BRI may serve as a simple, noninvasive screening tool that identifies individuals at high risk of cardiovascular events while simultaneously assessing MASLD risk, thereby optimizing screening strategies in resource‐limited primary care settings and supporting a more integrated focus on both liver and cardiovascular health. In addition, as composite markers of metabolic dysfunction, LAP and WTI have also been associated with metabolic syndrome and type 2 diabetes [39, 40, 41, 42]. Their application in MASLD screening may therefore facilitate the concurrent identification and management of these important metabolic risk factors. Although these novel anthropometric indices cannot replace comprehensive clinical assessment, they may provide practical alternatives for special populations and for primary care settings with limited access to imaging‐based diagnostic tools.

This study has several limitations. First, its cross‐sectional design precludes causal inference. Second, some variables were self‐reported and may therefore be subject to recall bias. Third, residual confounding cannot be excluded. Fourth, the number of participants with very high LAP values was limited. Finally, the diagnosis of MASLD and LF relied on CAP and LSM rather than histological confirmation, which may have introduced misclassification bias. Future prospective studies incorporating histological assessment are needed to validate these findings.

## 5 Conclusion

In summary, the findings of this study provide important evidence supporting the use of anthropometric indices for MASLD screening in primary care settings. Among the indices evaluated, BRI appears to be the most promising screening tool because of its simplicity, low cost, stability across different populations, and favorable clinical net benefit. Moreover, BRI demonstrated good diagnostic performance for identifying LF among individuals with MASLD. Although WTI and LAP also showed clinical utility, their overall performance was inferior to that of BRI.

## Funding

This work was supported by the Project 2024 Guangxi Appropriate Technology Development and Promotion of Traditional Chinese Medicine (grant No. GZSY2024060) and Technology Project of Guangxi Zhuang Autonomous Region for Disease Prevention and Control (grant No. GXJKKJ2026YB002).

## Ethics Statement

The portions of this study involving human participants, human materials, or human data were conducted by the Declaration of Helsinki and were approved by the NCHS Ethics Review Board.

## Consent

The patients/participants provided their written informed consent to participate in this study.

## Conflicts of Interest

The authors declare no conflicts of interest.

## Supporting information


**Table S1:** Weighted baseline characteristics of participants with or without LF in MASLD.
**Table S2:** Threshold and saturation effect analysis for the relationship between the nthropometric Indicator and MASLD.
**Table S3:** Weighted stratified associations between five novel anthropometric indicators‐related indices and LF in MASLD by age, sex, race, hypertension, and diabetes.
**Table S4:** Comparison of anthropometric indices between non‐MASLD individuals with and without CMRFs.
**Figure S1:** ROC curves of anthropometric indices for predicting MASLD in the sensitivity analysis restricted to non‐MASLD controls with CMRFs.
**Figure S2:** DCA of anthropometric indices for predicting MASLD in the sensitivity analysis restricted to non‐MASLD controls with CMRFs.

## Data Availability

The datasets supporting the findings of this study are publicly available in the NHANES database at https://wwwn.cdc.gov/nchs/nhanes/Default.aspx.
