# Supplementary material for: The Value of Five Novel Anthropometric Indicators in Evaluating MASLD and Liver Fibrosis in MASLD: A Cross‐Sectional Study
Source: JGH Open. 2026 Jul 8;10(7):e70435. doi: 10.1002/jgh3.70435 (PMC13343303; doi:10.1002/jgh3.70435)
Supplement: Supplementary file 1 — Table S1: Weighted baseline characteristics of participants with or without LF in MASLD. Table S2: Threshold and saturation effect analysis for the relationship between the nthropometric Indicator and MASLD. Table S3: Weighted stratified associations between five novel anthropometric indicators‐related indices and LF in MASLD by age, sex, race, hypertension, and diabetes. Table S4: Comparison of anthropometric indices between non‐MASLD individuals with and without CMRFs. Figure S1: ROC curves of anthropometric indices for predicting MASLD in the sensitivity analysis restricted to non‐MASLD controls with CMRFs. Figure S2: DCA of anthropometric indices for predicting MASLD in the sensitivity analysis restricted to non‐MASLD controls with CMRFs. [file JGH3-10-e70435-s001.docx]

**The Value of Five Novel Anthropometric Indicators in Evaluating MASLD and Liver Fibrosis in MASLD: A Cross-Sectional Study**

**Supplement tables**

Table S1 Weighted baseline characteristics of participants with or without LF in MASLD

| Variables | Total (n = 2726) | Non-LF(n = 2119) | LF (n = 607) | *P* |
| --- | --- | --- | --- | --- |
| AGE(years) | 49.78 ± 16.39 | 49.01 ± 16.55 | 52.50 ± 15.53 | <0.001 |
| GENDER (%) |  |  |  | 0.007 |
| Man | 1527 (56.02) | 1158 (54.65) | 369 (60.79) |  |
| Female | 1199 (43.98) | 961 (45.35) | 238 (39.21) |  |
| EDUCATION (%) |  |  |  | 0.774 |
| Less than high school | 391 (14.34) | 309 (14.58) | 82 (13.51) |  |
| More than high school | 2278 (83.57) | 1765 (83.29) | 513 (84.51) |  |
| Others | 57 (2.09) | 45 (2.12) | 12 (1.98) |  |
| MARITAL (%) |  |  |  | 0.587 |
| Married | 1674 (61.41) | 1297 (61.21) | 377 (62.11) |  |
| Unmarried | 547 (20.07) | 421 (19.87) | 126 (20.76) |  |
| Others | 505 (18.53) | 401 (18.92) | 104 (17.13) |  |
| RACE (%) |  |  |  | 0.010 |
| Mexican American | 423 (15.52) | 317 (14.96) | 106 (17.46) |  |
| Other Hispanic | 298 (10.93) | 228 (10.76) | 70 (11.53) |  |
| Non-Hispanic White | 1033 (37.89) | 795 (37.52) | 238 (39.21) |  |
| Non-Hispanic Black | 603 (22.12) | 466 (21.99) | 137 (22.57) |  |
| Other Race | 369 (13.54) | 313 (14.77) | 56 (9.23) |  |
| BMI (kg/m^2^) | 32.76 ± 7.26 | 31.56 ± 6.33 | 36.94 ± 8.62 | <0.001 |
| LSM(kPa) | 6.53 ± 5.59 | 4.92 ± 1.05 | 12.15 ± 9.78 | <0.001 |
| CAP (dB/m) | 307.43 ± 41.32 | 301.08 ± 38.12 | 329.59 ± 44.31 | <0.001 |
| Sleep hours(h/day) | 7.42 ± 1.56 | 7.42 ± 1.54 | 7.45 ± 1.62 | 0.604 |
| ALT(U/L) | 25.74 ± 18.23 | 23.94 ± 14.94 | 32.05 ± 25.76 | <0.001 |
| ALP(IU/L) | 78.92 ± 24.40 | 77.73 ± 22.60 | 83.11 ± 29.47 | <0.001 |
| AST(U/L) | 22.65 ± 12.96 | 21.45 ± 9.76 | 26.84 ± 19.99 | <0.001 |
| GRE（mg/dL） | 0.88 ± 0.34 | 0.87 ± 0.24 | 0.92 ± 0.56 | 0.029 |
| GLU(mg/dL) | 104.64 ± 38.61 | 101.53 ± 34.65 | 115.48 ± 48.56 | <0.001 |
| GGT(IU/L) | 36.99 ± 46.81 | 33.09 ± 38.96 | 50.63 ± 65.63 | <0.001 |
| TBIL (mg/dL) | 0.45 ± 0.25 | 0.44 ± 0.25 | 0.47 ± 0.26 | 0.066 |
| TC(mg/dL) | 189.63 ± 41.37 | 190.88 ± 40.99 | 185.26± 42.41 | 0.003 |
| HDL-C(mg/dL) | 50.39 ± 15.14 | 51.19 ± 15.00 | 47.62 ± 15.29 | <0.001 |
| BRI | 6.63 ± 2.44 | 6.21 ± 2.10 | 8.11 ± 2.92 | <0.001 |
| LAP | 83.71 ± 64.49 | 76.71 ± 58.16 | 108.18 ± 78.16 | <0.001 |
| WTI | 8.89 ± 0.58 | 8.84 ± 0.58 | 9.06 ± 0.56 | <0.001 |
| WWI | 11.28 ± 0.74 | 11.20 ± 0.73 | 11.56 ± 0.70 | <0.001 |
| ABSI | 8.20 ± 0.45 | 8.18 ± 0.45 | 8.28 ± 0.44 | <0.001 |
| FIB4 | 1.03 ± 0.69 | 0.97 ± 0.57 | 1.21 ± 0.98 | <0.001 |
| APRI | 0.25 ± 0.20 | 0.23 ± 0.13 | 0.31 ± 0.34 | <0.001 |
| Diabetes (%) | 536 (19.66) | 323 (15.24) | 213 (35.09) | <0.001 |
| Hypertension (%) | 614 (22.52) | 449 (21.19) | 165 (27.18) | 0.002 |

Continuous variables are shown as mean (SE) and their P value was calculated by linear regression model. Categorical values are shown as % (SE) and its P value was calculated by chi-square test.

Table S2 Threshold and saturation effect analysis for the relationship between the nthropometric Indicator and MASLD

| Variables | Outcome | Adjusted OR | 95% CI | *P* |
| --- | --- | --- | --- | --- |
| WTI | Model I |  |  |  |
|  | One line slope | 1.02 | 1.02-1.02 | <0.001 |
|  | Model II |  |  |  |
|  | < 8.33 | 0.54 | 0.36-0.82 | 0.004 |
|  | >= 8.33 | 2.05 | 1.35-3.12 | <0.001 |
| WWI | Model I |  |  |  |
|  | One line slope | 3.47 | 3.08-3.9 | <0.001 |
|  | Model II |  |  |  |
|  | < 10.49 | 0.82 | 0.54-1.25 | 0.355 |
|  | >= 10.49 | 4.13 | 2.65-6.44 | <0.001 |
| ABSI | Model I |  |  |  |
|  | One line slope | 2.25 | 1.9-2.68 | <0.001 |
|  | Model II |  |  |  |
|  | < 7.71 | 0.7 | 0.47-1.05 | 0.086 |
|  | >= 7.71 | 1.48 | 0.98-2.24 | 0.062 |
|  | *P* for LRT test |  |  |  |
| BRI | Model I |  |  |  |
|  | One line slope | 1.86 | 1.77-1.95 | <0.001 |
|  | Model II |  |  |  |
|  | < 3.6 | 0.27 | 0.17-0.42 | <0.001 |
|  | >= 3.6 | 2.44 | 1.59-3.77 | <0.001 |
| LAP | Model I |  |  |  |
|  | One line slope | 1.04 | 1.03-1.04 | <0.001 |
|  | Model II |  |  |  |
|  | < 24.28 | 0.4 | 0.26-0.61 | <0.001 |
|  | >= 24.28 | 2.92 | 1.89-4.5 | <0.001 |

Model I, linear analysis; Model II, non-linear analysis, LRT, Logarithmic likelihood ratio. The adjustment strategy is the same as the fully adjusted model.

Table S3 Weighted stratified associations between Five Novel Anthropometric Indicators-related indices and LF in MASLD by age, sex, race, hypertension, and diabetes.

| **Subgroup** | **WTI** | | **WWI** | | **ABSI** | | **BRI** | | **LAP** | |
| --- | --- | --- | --- | --- | --- | --- | --- | --- | --- | --- |
|  | **OR (95% CI)** | ***P* for interaction** | **OR (95% CI)** | ***P* for interaction** | **OR (95% CI)** | ***P* for interaction** | **OR (95% CI)** | ***P* for interaction** | **OR (95% CI)** | **p for interaction** |
| GENDER |  | 0.103 |  | 0.265 |  | 0.117 |  | 0.058 |  | 0.112 |
| Female | 2.13(1.46,3.13) |  | 1.70(1.36,2.13) |  | 0.79(0.57,1.11) |  | 1.37(1.29,1.46) |  | 1.01(1,1.01) |  |
| Man | 1.75(1.32,2.32) |  | 2.41(1.92,3.03) |  | 1.62(1.11,2.37) |  | 1.48(1.38,1.59) |  | 1.01(1,1.01) |  |
| AGE (years) |  | 0.581 |  | 0.049 |  | 0.006 |  | 0.862 |  | 0.479 |
| 18-45 | 1.89(1.29,2.75) |  | 2.68(2.01,3.57) |  | 1.87(1.16,3.02) |  | 1.46(1.35,1.58) |  | 1.01(1,1.01) |  |
| 45-60 | 1.83(1.22,2.76) |  | 1.82(1.38,2.39) |  | 0.82(0.53,1.26) |  | 1.41(1.3,1.53) |  | 1.01(1,1.01) |  |
| ≥60 | 1.78(1.19,2.66) |  | 1.82(1.38,2.42) |  | 0.97(0.64,1.47) |  | 1.39(1.27,1.51) |  | 1.01(1,1.01) |  |
| BMI(kg/m^2^) |  | 0.695 |  | 0.36 |  | 0.425 |  | 0.47 |  | 0.774 |
| <25 | 1.87(0.83,4.23) |  | 1.08(0.47,2.52) |  | 1.39(0.40,4.84) |  | 1.15(0.60,2.23) |  | 1.01(1,1.02) |  |
| 25-35 | 1.62(1.22,2.15) |  | 1.38(1.06,1.79) |  | 1.44(0.96,2.15) |  | 1.30(1.14,1.49) |  | 1.01(1,1.01) |  |
| >35 | 1.23(0.89,1.70) |  | 1.52(1.19,1.93) |  | 1.22(0.85,1.75) |  | 1.32(1.22,1.42) |  | 1.01(1,1.01) |  |
| RACE |  | 0.246 |  | 0.466 |  | 0.761 |  | 0.449 |  | 0.073 |
| Mexican American | 1.91(1.03,3.55) |  | 2.23(1.48,3.38) |  | 0.99(0.42,2.38) |  | 1.49(1.3,1.71) |  | 1.00(1,1.01) |  |
| Other Hispanic | 2.39(1.15,4.94) |  | 3.4(1.98,5.83) |  | 2.38(1.04,5.44) |  | 1.62(1.37,1.93) |  | 1.01(1,1.02) |  |
| Non-Hispanic White | 1.83(1.26,2.65) |  | 1.82(1.41,2.36) |  | 1.18(0.71,1.98) |  | 1.39(1.29,1.5) |  | 1.01(1,1.01) |  |
| Non-Hispanic Black | 2.21(1.38,3.55) |  | 1.91(1.39,2.63) |  | 0.83(0.55,1.25) |  | 1.35(1.24,1.47) |  | 1.01(1.01,1.02) |  |
| Other Race | 1.91(1.03,3.55) |  | 1.94(1.11,3.38) |  | 1.05(0.55,2.02) |  | 1.54(1.3,1.82) |  | 1.01(1,1.01) |  |
| HYPERTENSION |  | 0.118 |  | 0.009 |  | 0.169 |  | 0.037 |  | 0.315 |
| No | 2.05(1.58,2.66) |  | 2.16(1.8,2.6) |  | 1.07(0.8,1.43) |  | 1.45(1.38,1.54) |  | 1.01(1,1.01) |  |
| Yes | 1.26(0.8,1.98) |  | 1.71(1.24,2.37) |  | 1.09(0.66,1.79) |  | 1.32(1.21,1.44) |  | 1.00(1,1.01) |  |
| Diabetes |  | 0.39 |  | 0.688 |  | 0.03 |  | 0.073 |  | 0.837 |
| No | 1.79(1.38,2.33) |  | 1.94(1.62,2.33) |  | 1.14(0.85,1.52) |  | 1.38(1.31,1.46) |  | 1.01(1,1.01) |  |
| Yes | 2.09(1.35,3.23) |  | 2.21(1.58,3.09) |  | 0.88(0.54,1.45) |  | 1.49(1.35,1.65) |  | 1.01(1,1.01) |  |

Table S4 Comparison of anthropometric indices between non-MASLD individuals with and without CMRFs

| Variables | Total (n = 1997) | non-MASLD without CMRFs  (n = 553) | non-MASLD with CMRFs  (n = 1444) | *P* |
| --- | --- | --- | --- | --- |
|  |  |  |  |  |
| BRI, Mean ± SD | 4.16 ± 1.73 | 2.62 ± 0.58 | 4.74 ± 1.66 | <0.001 |
| LAP, Mean ± SD | 34.70 ± 33.07 | 13.50 ± 7.13 | 42.82 ± 35.43 | <0.001 |
| WTI, Mean ± SD | 8.30 ± 0.54 | 7.93 ± 0.36 | 8.44 ± 0.53 | <0.001 |
| WWI, Mean ± SD | 10.53 ± 0.82 | 9.91 ± 0.56 | 10.76 ± 0.78 | <0.001 |
| ABSI,Mean ± SD | 7.93 ± 0.49 | 7.73 ± 0.40 | 8.01 ± 0.50 | <0.001 |

| 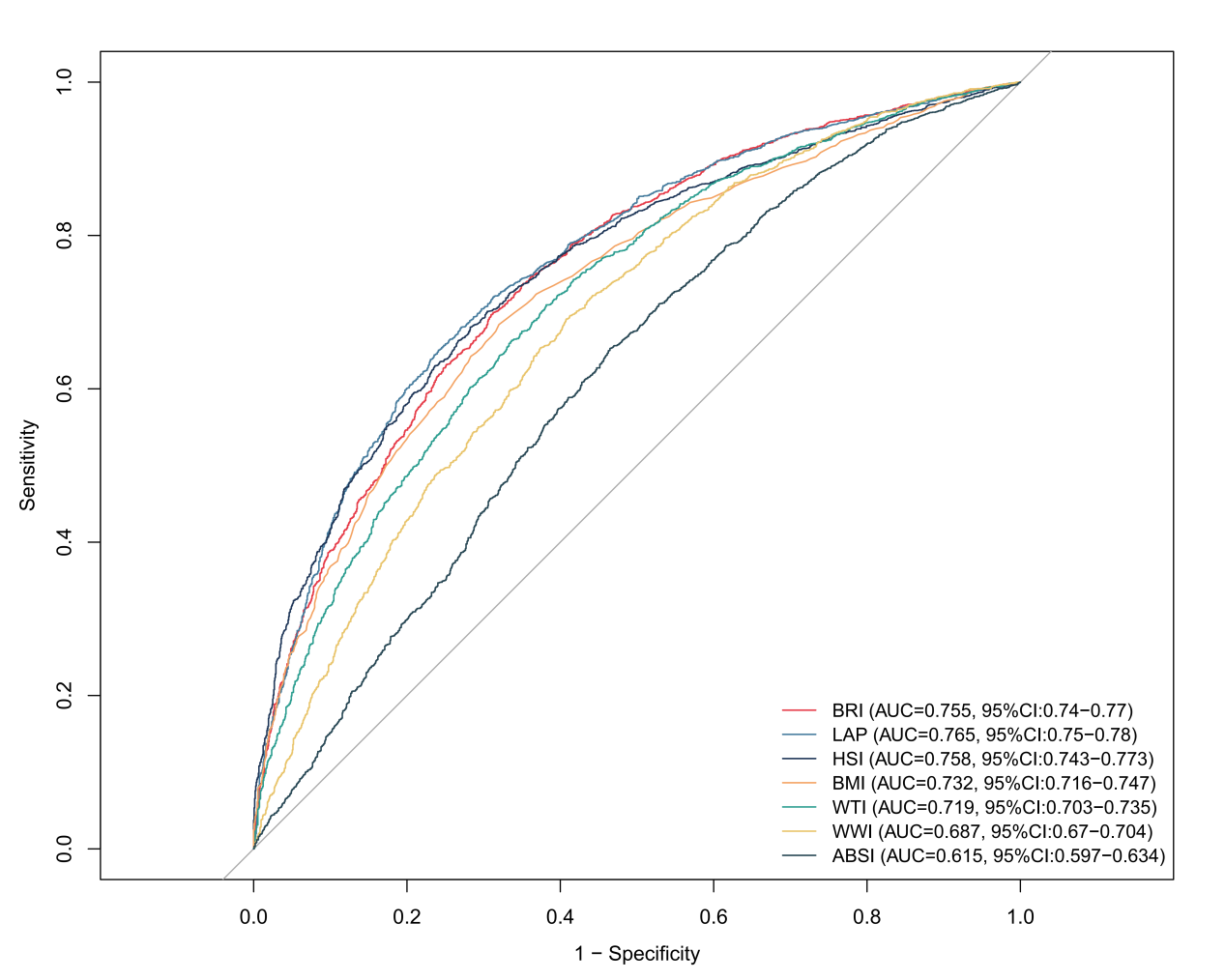 |
| --- |

Figure S1. ROC curves of anthropometric indices for predicting MASLD in the sensitivity analysis restricted to non-MASLD controls with CMRFs.

| 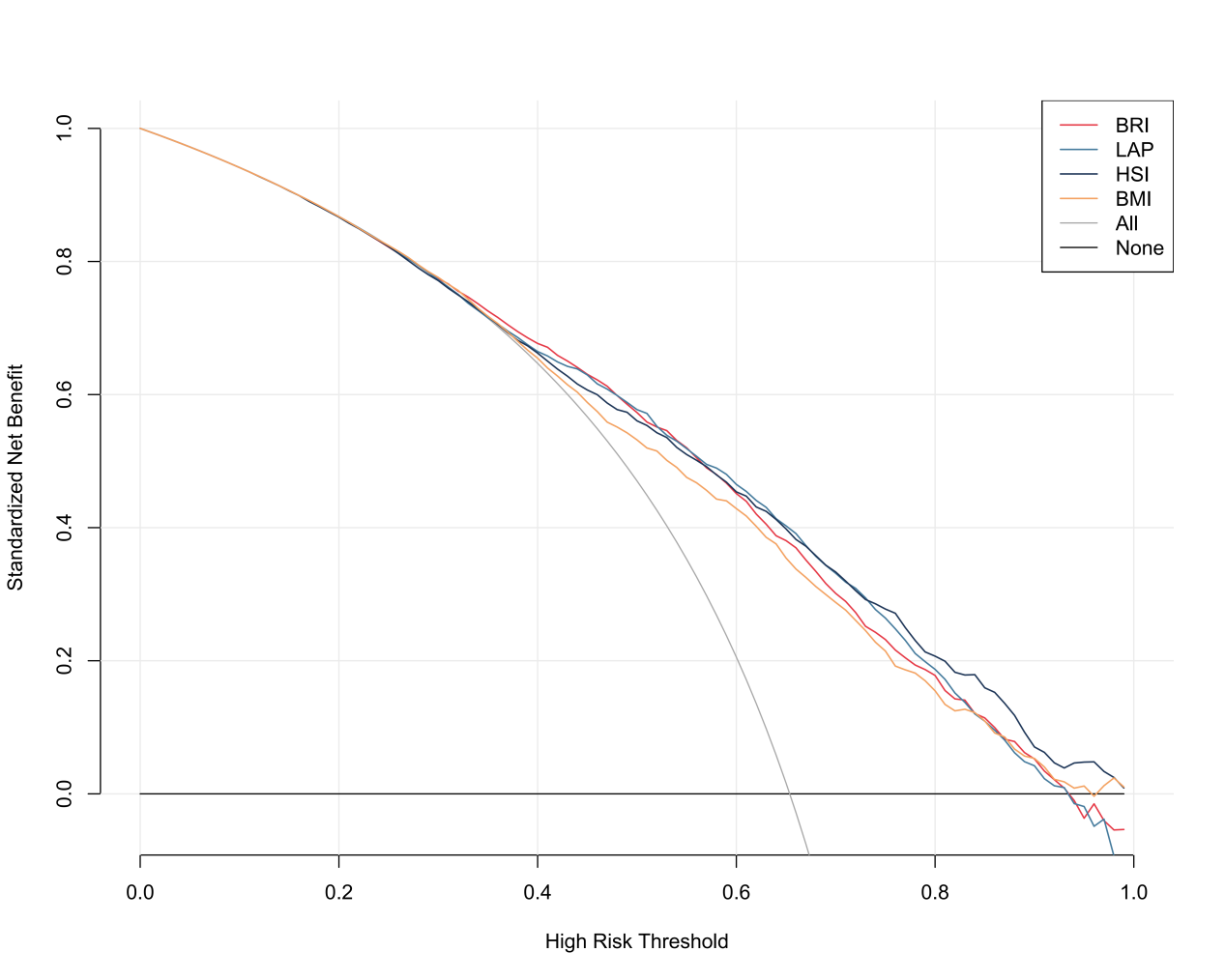 |
| --- |

Figure S2. DCA of anthropometric indices for predicting MASLD in the sensitivity analysis restricted to non-MASLD controls with CMRFs.
